# Hepatitis B virus middle surface antigen loss promotes clinical variant persistence in mouse models

**DOI:** 10.1080/21505594.2021.1999130

**Published:** 2021-11-21

**Authors:** Junyu Lin, Jing Li, Peilin Xie, Yue Han, Demin Yu, Jia Chen, Xinxin Zhang

**Affiliations:** Department of Infectious Diseases, Research Laboratory of Clinical Virology, National Research Center for Translational Medicine (Shanghai), Ruijin Hospital, Shanghai Jiao Tong University School of Medicine, Shanghai, China

**Keywords:** hepatitis B virus, middle surface antigen, chronic infection, start codon mutation, cytokines

## Abstract

Hepatitis B virus (HBV) middle surface antigen (MHBs) mutation or deletion occurs in patients with chronic HBV infection. However, the functional role of MHBs in HBV infection is still an enigma. Here, we reported that 7.33% (11/150) isolates of CHB patients had MHBs start codon mutations compared with 0.00% (0/146) in acute hepatitis B (AHB) patients. Interestingly, MHBs loss accounted for 11.88% (126/1061) isolates from NCBI GenBank, compared with 0.09% (1/1061) and 0.00% (0/1061) for HBV large surface antigen (LHBs) loss and HBV small surface antigen (SHBs) loss, respectively. One persistent HBV clone of genotype B (B56, MHBs loss) from a CHB patient was hydrodynamically injected into BALB/c mice. B56 persisted for >70 weeks in BALB/c mice, whereas B56 with restored MHBs (B56^M+^) was quickly cleared within 28 days. Serum cytokine assays demonstrated that CXCL1, CXCL2, IL-6 and IL-33 were significantly increased during rapid HBV clearance in B56^M+^ mice. Furthermore, the enhancers and promoters of B56 were proved to be required for B56 persistence in mice. Ablating MHBs expression improved the persistence of a new clone (HBV1.3, genotype B) which was recreated by using enhancers and promoters of B56. These data demonstrated that MHBs deletion can promote the persistence of specific HBV variants in a hydrodynamic mouse model. MHBs re-expression restored a rapid clearance of HBV, which was accompanied by cytokine responses including the elevation of CXCL1, CXCL2, IL-6 and IL-33.

## Introduction

The hepatitis B virus (HBV) genome (~3.2 kb) consists of partially double-stranded, relaxed circular DNA (rcDNA) that is extremely compactly organized with overlapping open reading frames encoding viral proteins. In addition, transcriptional regulatory elements such as promoters, enhancers, and the polyadenylation and encapsidation signals overlap with coding sequences [1]. After hepatocyte attachment and entry, HBV rcDNA is converted to covalently closed circular DNA (cccDNA) in the nucleus, which serves as the template for viral transcription. Four promoters (S promoter 1, S promoter 2, Core promoter, and X promoter) in concert with two enhancers (Enhancer I and Enhancer II) control HBV transcription [2–4].

The HBV envelope contains a lipid bilayer and three glycoproteins, referred to as the large, middle, and small hepatitis B surface antigens (LHBs, MHBs, and SHBs, respectively). The S, M, and L proteins are encoded within one open reading frame of the viral genome using three different in-frame translational start codons. They share the same S domain of HBsAg in the C-terminal region and only differ in length because of their N-terminal regions; the pre-S1 domain is present exclusively in the L protein and the pre-S2 domain occurs in both L and M proteins [3]. SHBs are the predominant component of infectious virions and subviral particles (SVPs), with LHBs and MHBs as minor components. The loss of SHBs completely abolishes the secretion of new virions. In vitro studies showed that LHBs with the preS1 domain that includes the high affinity attachment site to the HBV receptor, sodium taurocholate co-transporting polypeptide (NTCP) [5], and facilitates the envelopment of the core particles. Unlike its absence in avian hepadnavirus, the synthesis of MHBs is observed within all orthohepadnaviruses, which might reflect a specific function of MHBs in HBV infection [6,7]. MHBs expression is not essential for HBV replication, virion morphogenesis, or HBV or hepatitis D virus infectivity [8–10]. It has been demonstrated clinically that the detection of HBV variants defective in preS2 region is not a rare event in patients with chronic HBV infection [11,12]. Furthermore, the quantification of LHBs and MHBs was described as a novel tool to identify inactive HBV carriers and better predict therapeutic response than total HBsAg concentration [13,14]. However, the biological role of MHBs remains mostly elusive.

HBV usually causes self-limiting infection in immunocompetent adults, but establishes chronic infection in a minority of adults and in most maternally infected infants[1]. Viral and host factors determining clearance versus persistence are not yet fully elucidated, partially due to scarcity of ideal animal models of HBV persistence. Hydrodynamic injection (HDI) is a high-volume tail vein injection readily suited for the delivery into hepatocytes of plasmid encoding replication competent HBV, but the duration of HBV gene expression is often transient in immunocompetent adult mice, manifesting as acute infections [15,16]. Although HBV cannot “infect” murine hepatocytes, the plasmid encoding an overlength of HBV DNA is transfected into a proportion of hepatocytes and mimics the natural cccDNA as a transcriptional template [17]. At present, sustained expression of the HBV genome in mouse models mostly depends on immunodeficiency mice or transduction effects of the viral vector such as adenovirus associated viral vector rather than solely on HBV sequence [18,19], which may complicate studies on the mechanism of chronic HBV infection. To overcome this, HBV persistence based on a non-viral vector in hydrodynamically transfected mice is a feasible alternative. Generally, HBV DNA based on non-viral vector (plasmid pUC18-HBV1.3) is quickly cleared in hydrodynamically transfecting mice. Thus, we employed an HBV genotype B isolate (B56) which was isolated from the serum of a chronic hepatitis B patient and persisted for 33 weeks in ~50% of BALB/c mice and C57bl/c mice [20].

In the present study, new HBV clones containing a 1.3 unit-length HBV genome were generated using specific mutations or transcriptional elements of B56. The different mutants were studied in Huh7 cells and in a BALB/c mouse model for viral gene expression, viral replication, and the associated cytokine expression in mice. This work provides insight on the functional role of MHBs in the persistence of HBV infection.

## Materials and methods

### Sequence data

In total, 146 human HBV genome sequences from 10 acute hepatitis B (AHB) patients and 150 sequences from 10 chronic hepatitis B (CHB) patients, previously established as nonrecombinant, were selected [21]. B56 as a reference sequence was compared with 1061 HBV full-length genotype B sequences from NCBI GenBank to identify variations at the nucleotide level. Sequencing quality and data manipulations were analyzed using Codon Code Aligner v9.0.1 (CodonCode Corporation, Dedham, MA, USA).

### HBV strains, plasmid constructs and antibodies

B56 (#AF100309.1) and B6 (#KR152339) were isolated from serum of CHB patients respectively, and other strains (A1.3, adw, #AP007263.1; C1.3, adr, #KX449554.1; and D1.3, ayw, #V01460;) were chemically synthesized. pCMV-HBV1.1 carrying a 1.1-fold length of B56 genomic DNA under the cytomegalovirus (CMV) promoter was given by Dr. Zhongliang Shen (Fudan University). HDI plasmids were constructed by cloning an HBV genome with a ~1.3-fold increase in length into pUC18. 56A1.3, 56B1.3, 56C1.3, 56D1.3 and B6-PLUS were chemically synthesized. The B56^M+^, B6^M-^, B56^M+/L-^, B56^M+/S-^, B56^LMS-^, 56A1.3^M-^, 56B1.3^M-^, 56C1.3^M-^, 56D1.3^M-^ and B6-PLUS^M+^ plasmids were constructed by polymerase chain reaction, using a mismatched primer that changed GTG to ATG or ATG to GTG in the start codon respectively. The firefly luciferase reporter constructs of HBV promoters/enhancers were described previously [22]. IFN-β-Luc and NF-κB-Luc reporter plasmids were gifted from Dr. Zhongliang Shen of Fudan University, China. AP-1-Luc (#11535ES03) and ISRE (interferon stimulated response element)-Luc (#11518ES03) reporter plasmids were purchased from Yeasen (Shanghai, China). Polyclonal rabbit anti-hepatitis B virus core antigen (HBc) antibody (B0586) was purchased from Dako (Glostrup, Denmark). Polyclonal rabbit anti-HBsAg antibody (NB100-62,652) was purchased from Novus Biological (Littleton, CO, USA). Anti-Rabbit IgG (H + L) cross-adsorbed secondary antibody (A0208) was purchased from Byotime (Shanghai, China).

### Mice and hydrodynamic injection

All animal protocols were approved by the Animal Care and Use Committee of Ruijin Hospital of Shanghai Jiao Tong University. Male 6–7-week-old specific pathogen-free BALB/c mice were purchased from Experimental Animal Center. The HBV genomic DNA cloned in the plasmid vector was used for hydrodynamic injection. Briefly, 10 μg of HBV DNA in 2 ml of phosphate-buffered saline (PBS) was injected into male mice via the tail vein within 5–8 s. Twenty-four hours later, serum was collected for HBsAg and HBeAg analysis.

### HBV serological assays

HBsAg, HBeAg and anti-HBsAg antibodies were analyzed using the Abbott Architect immunoassay system (Abbott Laboratories). Mouse serum was diluted 8-fold with PBS, and 250 ml of diluted sample was used for the assay.

### Cell culture and transfection

Huh7 cells were maintained in Dulbecco’s modified Eagle’s medium (Gibco) supplemented with 10% fetal bovine serum, 50 mg/ml penicillin, 50 mg/ml streptomycin, and 100 mg/ml kanamycin at 37°C in 5% carbon dioxide. Huh7 cells were transfected with plasmids using polyetherimide (PEI) (B600070, proteintech^TM^) as directed by the manufacturer.

### HBV infection

Huh7 cells were transfected with plasmid B56 or B56^M+^, respectively. HBV particles were concentrated from the purified supernatant by conducting overnight precipitation with 10% PEG 8000 and centrifugation at 4°C for 1 h at 16,000 g. The pellet was re-suspended in the Opti-MEM medium supplemented with 2.5% DMSO at a concentration of 10^9^ genome equivalents (GE) per milliliter. HBV inoculum concentration was quantified using real-time PCR by following the protocol of HBV DNA detection. HepG2-NTCP cells were inoculated with HBV at 1,000 GE per cell in a complete medium containing 2.5% DMSO, 4% PEG8000 for 12 h at 37°C and 5% CO2. Cells were washed with PBS 5 times and added with fresh culture medium. Cell culture medium was changed every 2–3 days and the supernatants were harvested at 8 days post-inoculation.

### Southern and Northern blot analysis

HBV DNA in core particles was isolated and analyzed by southern blotting using the DIG-labeled HBV RNA probe prepared using the DIG Northern Starter Kit (Roche Diagnostics) as described previously [22].

10 μg of total RNA was subjected to formaldehyde-1% agarose gel and transferred onto a nylon membrane. RNA transcripts were detected with a DIG-labeled HBV RNA probe according to the manufacturer’s instructions as described previously [22].

### Reporter gene assays

Cells were transiently transfected with promoter-driven firefly luciferase reporter plasmids and the constitutive *Renilla* luciferase reporter vector pRL-TK (Promega) using transfection kit according to the manufacturer’s instructions. At 48 h post-transfection, cell lysates were collected and analyzed for luciferase activity using the Promega Dual-Luciferase assay system according to the manufacturer’s instructions. Luciferase activity of the relative firefly was normalized to luciferase activity of the relative *Renilla*.

### Immunohistochemical analysis for HBcAg and sirius red staining

Mouse liver tissues were formalin fixed and paraffin embedded and then sectioned for HBcAg. The liver sections were deparaffinized and rehydrated and incubated with primary antibody anti-HBcAg overnight at 4°C, followed by incubation with horseradish peroxidase-conjugated anti-rabbit-IgG (H + L). A further tissue section was stained with sirius red to examine the collagen production area. The histological and staining images were captured and visualized using NDPview.2 (Hamamatsu Photonics K.K., Japan).

#### Western blot analysis

Cell lysates were prepared using the cell lysis buffer (P0013; Beyotime, Shanghai, China). Equal amounts of total protein were loaded onto 8%-12% polyacrylamide gels, separated by sodium dodecyl sulfate-polyacrylamide gel electrophoresis (SDS-PAGE), and transferred to a nitrocellulose membrane (Roche). The membrane was blocked with 5% skimmed milk for 2 hours at room temperature and then incubated overnight with the primary antibody. Next, the membranes were washed three times with phosphate-buffered saline tween (PBST) and incubated with a horseradish peroxidase-conjugated secondary antibody for 3–4 h. After further washing with PBST, the signal intensities of the membranes were visualized using an ECL^TM^ western blotting system.

### Serum cytokine assays

Serum samples were prepared by centrifugation at 300 g for 15 minutes and stored at −80°C. A Mouse Magnetic Luminex assay (R&D Systems, USA) was used by the manufacturer’s instructions. The complete panel was screened for the expression of the following: CXCL1, CXCL2, IL-2, IL-4, IL-6, IL-10, IL-12, IL-17, IL-33 and TNF-α. IL-21 was measured by ELISA (FineTest, Wuhan, China).

### Statistical analyses

Data are presented as the mean ± SD of at least three independent experiments, and p values were determined by two-tailed Student’s t tests using GraphPad Prism 7.04 software (San Diego, CA, USA). *P* < 0.05 was considered statistically significant (ns, there was no significant difference, * *P* < 0.05, ** *P* < 0.01, and *** *P* < 0.001).

## Results

### MHBs loss due to start codon mutations was frequently associated with CHB patients

**Figure 1.**
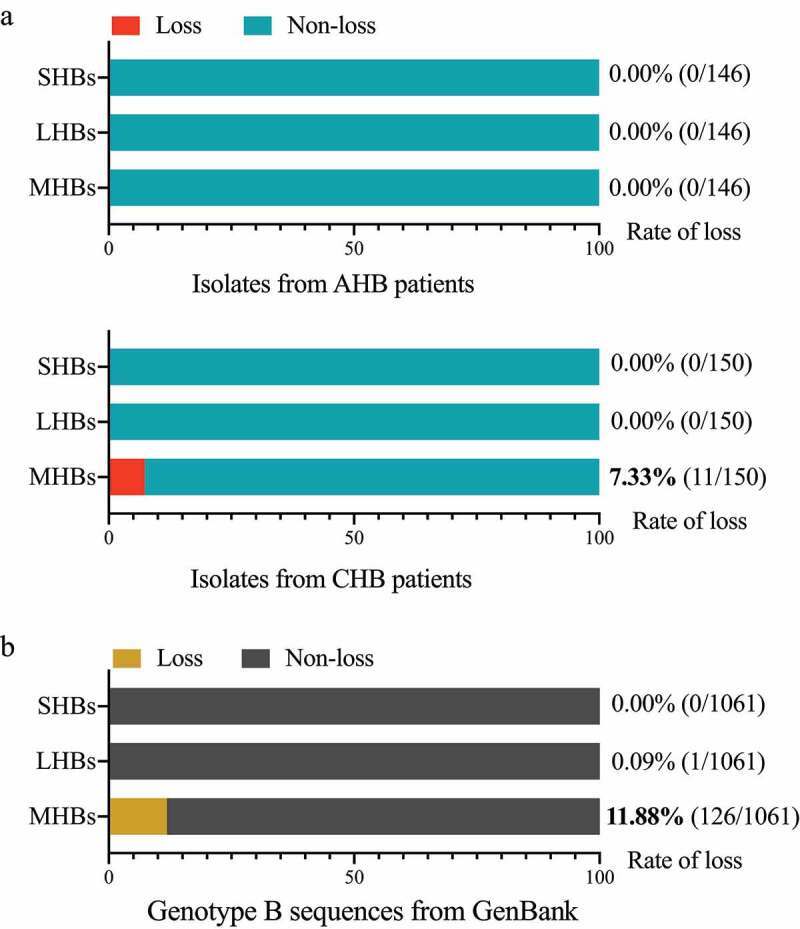
**MHBs Loss is Frequently Associated with Chronic Hepatitis B (CHB) Patients**. The rates of variants with deficiency in LHBs, MHBs, and SHBs synthesis induced by mutations in the start codon (ATG) are represented in the schematic diagram. (a) The rates of isolates with deficiency in LHBs, MHBs, and SHBs from acute hepatitis B (AHB) patients (n = 10) versus CHB patients (n = 10), and (b) NCBI GenBank (1061 complete sequences of HBV genotype B)

To investigate the proportion of MHBs-deficiency variants with start codon mutations, we analyzed HBV full-length sequences from 10 treatment-naïve CHB patients (146 clones) and 10 AHB patients (150 clones) (details shown in Table 1 and Table S1). The results showed that the ratio of MHBs loss was 7.33% (11/150) in CHB set from 3 patients, compared with 0.00% (0/150) in AHB set. None of the patients had LHB or SHB loss (Figure 1a). Furthermore, we performed an extensive analysis of HBV complete isolates of genotype B from the NCBI GenBank database (details shown in Table S2). As shown in Figure 1b, the rate of variants with start codon mutations in the preS2 region was 11.88%, implying that 126 of 1061 isolates lost MHBs synthesis. In contrast, the prevalence of LHBs start codon mutants was 0.09% (1/1061) and that of SHB was 0.00% (0/1061).

**Table 1.** Characterization of HBV infected patients

	AHB (n = 10)	CHB (n = 10)
Age (y)	40.7 ± 2.6	35.8 ± 4.3
Gender, Males, n (%)	9 (90.0%)	6 (60.0%)
ALT (IU/L)	1608.5 ± 199.3	427.9 ± 228.1
HBeAg positive, n (%)	10 (100.0%)	9 (90.0%)
HBeAg negative, n (%)	4 (40.0%)	1 (10.0%)
HBV DNA (log10 IU/mL)	5.9 ± 0.5	7.3 ± 0.4
HBV Genotype (B/C)	4/6	6/4
LHBs Loss due to start codon mutation		
Patients/all patients	0/10	0/10
Clones/all clones	0/146	0/150
MHBs Loss due to start codon mutation		
Patients/all patients	0/10	3/10
Clones/all clones	0/146	11/150
SHBs Loss		
Patients/all patients	0/10	0/10
Clones/all clones	0/146	0/150
Accession IDs of Sequences	KU963799-KU963944	KU964079-KU964228

ALT: Alanine aminotransferase; LHBs: Large hepatitis B surface proteins; MHBs: Middle hepatitis B surface proteins; SHBs: Small hepatitis B surface proteins

### Hydrodynamic injection of a MHBs deficient clone (B56) establishes HBV persistence in BALB/c mice

**Figure 2.**
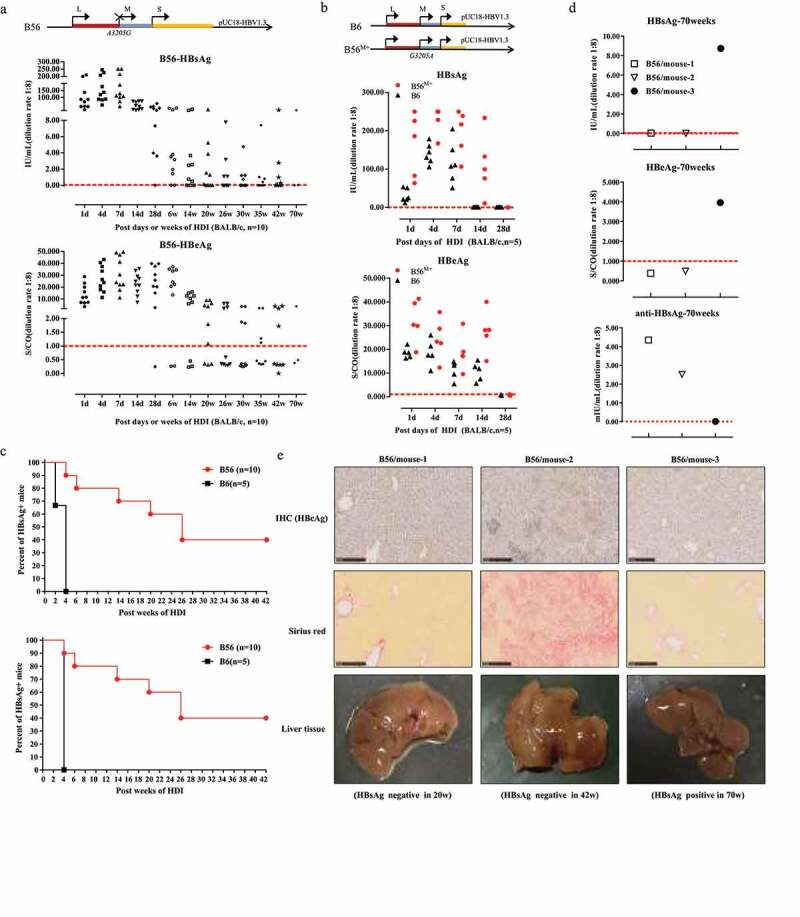
**HBV Plasmid B56 Transfected via Hydrodynamic Injection (HDI) Displays Persistence in BALB/c Mice for More than 70 Weeks**. BALB/c mice were injected hydrodynamically via the tail vein with 10 μg of HBV plasmid pUC18-HBV1.3 within 5–8 s. The titers of HBsAg and HBeAg from HDI-mice were determined at the indicated time points with a 1:8 dilution of sera with phosphate-buffered solution. The levels of HBsAg and HBeAg in B56 mice (n = 10) (a) and B56^M+^ mice (n = 5) and B6 mice (n = 5) (b) were measured at different time points after pHBV1.3 injection. Schematic illustrations of the genomic (B56, B56^M+^, B6) are presented and start codons of the LHBs, MHBs and SHBs are marked by arrows. Cutoff thresholds of determination were 0.05 IU/ml for HBsAg and 1.000 S/CO for HBeAg, which are represented by dotted lines. (c) The positive rates of serum HBsAg and HBeAg in mice were analyzed by Kaplan–Meier analysis, and the difference was statistically significant (*P* < 0.001). (d) The levels of HBsAg, HBeAg, and anti-HBsAg were detected in three B56 mice surviving at 70 weeks. (e) Liver sections taken from B56 HDI mice were stained for HBcAg and Sirius red (scale bars: 250 μm). IHC, immunohistochemistry

To test the duration of persistence of the B56 strain (genotype B, MHBs loss) in vivo, the plasmid harboring a replication competent 1.3-unit length of the HBV genome, plasmid pUC18-HBV1.3-B56, was injected via the tail vein into BALB/c mice (n = 10) via HDI. There were only three B56 mice survived for 70 weeks and the serum HBsAg and HBeAg levels were detected (Figure 2a). As an acute infection control, all BALB/c mice injected with pUC18-HBV1.3-B6 (B6, another genotype B clinical isolate) showed rapid clearance of serum HBsAg and HBeAg within 28 days (Figure 2b).

Importantly, mice injected with plasmid B56^M+^ with restored MHBs expression also displayed quick clearance within 28 days (Figure 2b), suggesting that MHBs loss is critical for B56 persistence. Our analysis results shown that B56 persistence for 42 weeks in 4 (40%) of HDI-mice (n = 10), as compared to the negative detection of antigens in all B6 mice (Figure 2c), demonstrated that B56 HDI-mice is a chronic HBV infection model.

Among these B56 HDI-mice, three mice survived for 70 weeks: mouse-1 and mouse-2 cleared HBsAg and HBeAg, accompanied by significant levels of anti-HBsAg, and mouse-3 showed detectable HBsAg with negative anti-HBsAg (Figure 2d). In addition, liver tissues of these three mice were subjected to immunohistochemistry and sirius red staining to detect HBcAg and liver fibrosis, respectively. The analysis results revealed that HBcAg, an important marker for HBV replication, was detected negative in these three mice and a sharp increase in collagen fibers in the liver of mouse-2, in which serum HBsAg was negative at week 42 (Figure 2e).

### Designed as the HBV1.3 clone, is required for B56 persistence in HDI mice

**Figure 3.**
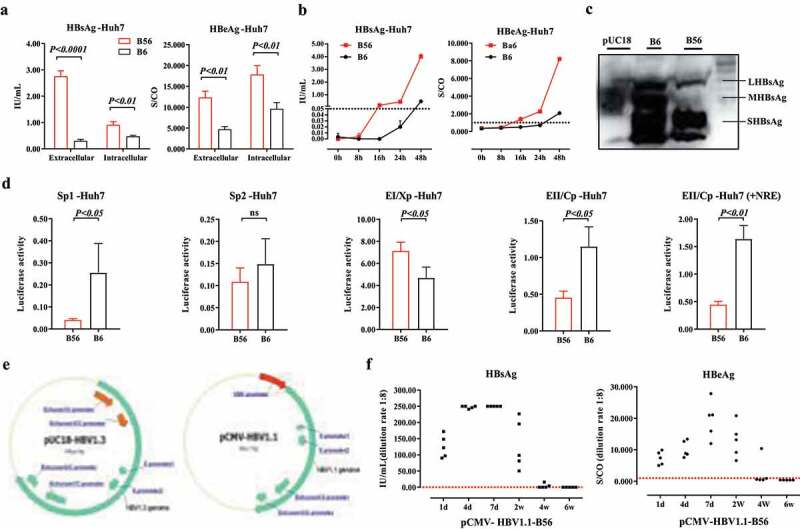
**B56 Persistence in BALB/c Depends on a 1.3-fold Length Genetic Recombination**. (a) At 48 h post-transfection, Huh7 cell culture supernatants were collected and cells were lysed and analyzed for extracellular and intracellular levels of HBsAg and HBeAg. (b) Cell culture supernatants were collected at indicated time points and analyzed for HBsAg and HBeAg levels. (c) The expression of HBsAg in Huh7 cells was detected by western blot analysis. (d) Promoter-driven firefly luciferase reporter plasmids (Sp1, Sp2, EnI/Xp, EnII/Cp, and EnII/Cp+NRE) were transfected into Huh7 cells. Cultured cells were lysed and assessed for luciferase activity. Sp1, S promoter1; Sp2, S promoter2; EnI/Xp, Enhancer I/X promoter; EnII/Cp, Enhancer II/Core promoter; NRE, negative regulative element. (e) Plasmid profiles of pUC18-HBV1.3 and pCMV-HBV1.1. (f) 10 μg of pCMV-HBV1.1 was transfected into BLAB/c mice (n = 5) by HDI. Sera were collected for analyses of HBsAg and HBeAg levels

To detect viral gene expression levels of B56 and B6 in vitro, pUC18-B56 and B6 were transiently transfected into Huh7 cells. Forty-eight hours post-transfection, cell culture supernatants were collected and cells were lysed to detect extracellular and intracellular levels of HBsAg and HBeAg. Results demonstrated that both the expression and secretion levels of B56 HBsAg were far above the levels of B6 (Figure 3a). Consequently, HBsAg and HBeAg in B56 were detectable, at 8 hours post-transfection, earlier than B6 (Figure 3b). In addition, the loss of MHBs expression (A3205G mutation) in B56 was confirmed by western blotting (Figure 3c). To further investigate the regulatory elements of HBV gene expression, the firefly luciferase reporter constructs of HBV promoters/enhancers were transfected into Huh7 cells. Results showed that except for Enhancer I/Core promoter (EnI/Cp) and S promoter 2 (Sp2), luciferase activities conferred by the S promoter1 (Sp1), Enhancer II/X promoter (EnII/Xp), and EnII/Xp together with the negative regulative element (NRE) of B56 were all lower than those with B6 (Figure 3d).

Interestingly and importantly, HDI with pCMV1.1-B56 in which gene expression was initiated by the potent CMV promoter (Figure 3e) did not cause B56 strain to persist in BALB/c mice (Figure 3f). The 1.3 × pHBV clone (pHBV1.3) consists of more than the full genome size of HBV with two copies of the enhancer/promoter, respectively located at the 5ʹ end and the 3ʹ end of the genome (Figure 3e). These results support the hypothesis that the persistence of B56 depends on a 1.3-fold genomic length, which results in complex modulation of HBV gene expression, regulated by HBV enhancers and promoters. Besides, although B56 exhibited relatively lower Sp1 and EnII/cp activities, its higher activity of EnI/Xp had the dominant effect in the regulation of pHBV1.3 gene expression [23], which guarantee the dramatically higher expression levels of HBsAg and HBeAg by comparing with B6.

### MHBs deficiency promotes persistence of the genotype B clone using the specific promotors and enhancers of B56

**Figure 4.**
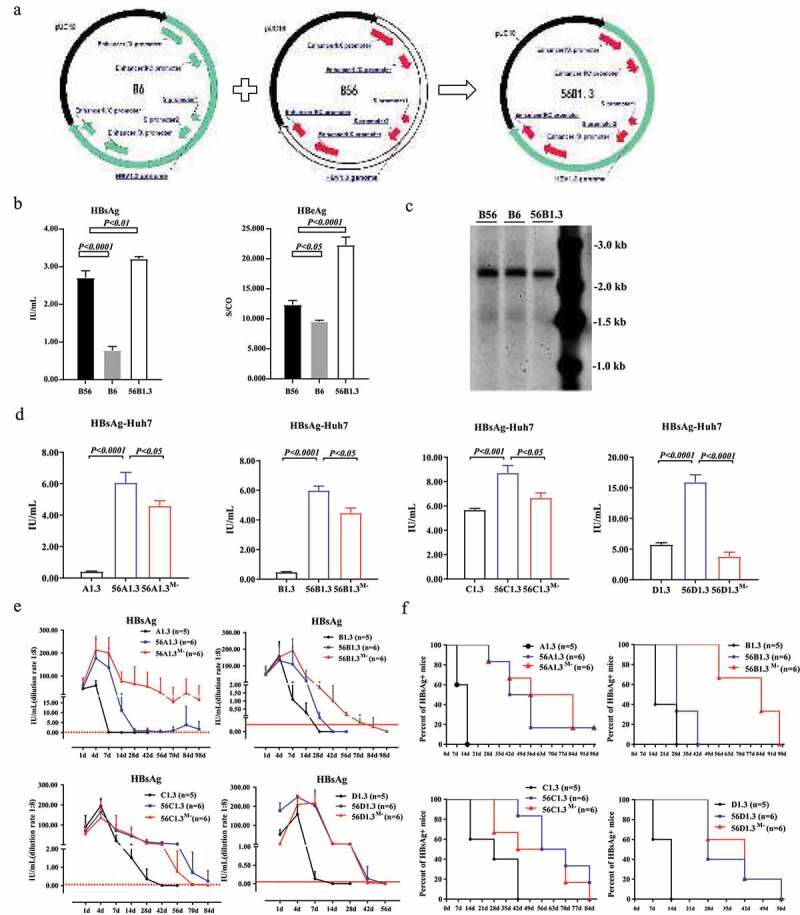
**B56 Persistence in Mice is Associated with its Specific Promoters and Enhancers**. (a) Plasmid profiles of B56, B6, and 56B1.3. The enhancers and promoters of B6 were correspondingly replaced with the enhancers and promoters of B56 to generate a new pHBV1.3 plasmid, 56B1.3. (b) The expression levels of HBsAg and HBeAg in transfected Huh7 culture. (c) pHBV1.3 replication intermediates were examined by southern blotting. (d) 56A1.3, 56B1.3, 56C1.3, and 56D1.3 were constructed respectively based on pUC18-HBV1.3A (A1.3), pUC18-HBV1.3B (B1.3, B6), pUC18-HBV1.3 C (C1.3), and pUC18-HBV1.3D (D1.3). The superscript M- represents the loss of MHBs. Forty-eight hours post-transfection of plasmids in Huh7 cells, the levels of HBsAg were measured. (e) The levels of HBsAg and (f) positive rates of serum HBsAg in pHBV1.3-HDI mice at the indicated time points were analyzed

Sustained gene expression of B56 was ultimately related with the regulation of the promoters and enhancers. To confirm the contributions of these elements to the persistence of B56, we used the promoters and enhancers of B56 to replace these elements in B6, generating a new pHBV1.3 plasmid 56B1.3 (Figure 4a). The HBsAg level of 56B1.3 was approximately equivalent to that of B56, whereas the HBeAg level was relatively higher than that in B56 (Figure 4b). Furthermore, there were no differences in replication levels among B56, B6, and 56B1.3 (Figure 4c). To explore promoter and enhancer regulation of B56 in other genotypes, pHBV1.3-A, pHBV1.3-C, and pHBV1.3-D were reconstructed to generate 56A1.3, 56C1.3, 56D1.3, similar to 56B1.3 construct. Meanwhile, 56A1.3^M-^, 56B1.3^M-^, 56C1.3^M-^, and 56D1.3^M-^ lacking preS2 start codons were created to further investigate the function of MHBs. As shown in Figure 4d, the promoter and enhancer of B56 could improve the expression of HBsAg in all four genotypes, while MHBs loss reduced the secretion levels of HBsAg. In HDI mice, the promoter and enhancer of B56 also helped all four genotype clones to delay HBsAg clearance (Figure 4e, f). The positive serum HBsAg rates in pHBV1.3-HDI mice showed that the persistence of serum HBsAg with 56B1.3^M-^ was significantly superior to that with 56B1.3 and B6 over time (Figure 4f), which suggested that the loss of MHBs potentially helped the HBV genotype B strain to achieve persistence in mice.

### Reduction or even deletion of total HBsAg does not influence B56 persistence in a mouse model

**Figure 5.**
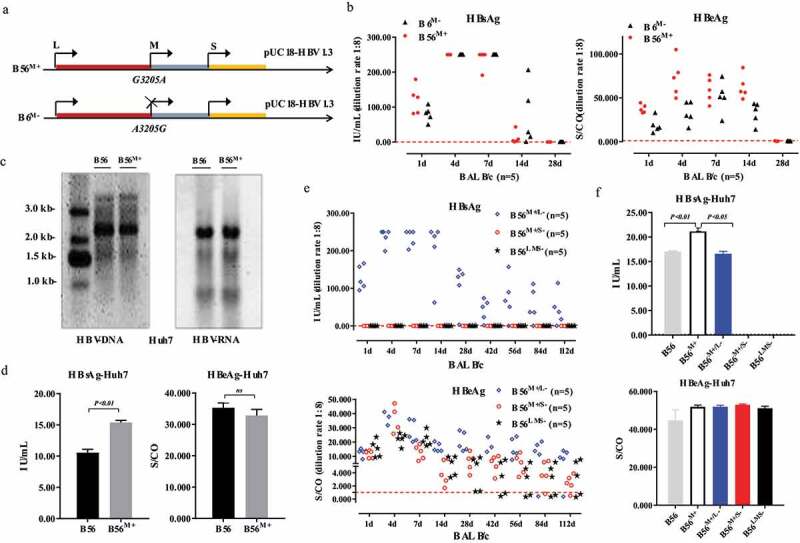
**Total HBsAg Deletion Does not Influence B56 Persistence in BALB/c Mice**. (a) Schematic illustrations of the genomic (B56^M+^, B6^M-^). (b) The HBsAg and HBeAg levels in mice induced by hydrodynamic injection of B56^M+^ and B6^M-^. (c) HBV DNA replicative intermediates from intracellular core particles and HBV RNA extracted from transfected Huh7 cells were detected by southern and northern blotting, respectively. (d) Huh7 cellular supernatants were collected for the detection of HBsAg and HBeAg. (e) HBV plasmids B56^M+/L-^ (expressed MHBs but LHBs loss), B56^M+/S-^(expressed MHBs but SHBs loss) and B56^LMS-^ (total HBsAg deletion). Sera from HDI mice were collected and analyzed for HBsAg and HBeAg. (f) HBV plasmids, as indicated, were transfected into Huh7 cells, and HBsAg and HBeAg were measured 48 h post-transfection

As shown in Figure 5a, the preS2 start codon was reinstated in B56 to create B56^M+^, which displayed rapid clearance in BALB/c mice with similar acute outcomes in B6^M-^ (MHBs loss) mice (Figure 5b). To further investigate the effect of MHBs on HBV gene expression in vitro, B56 and B56^M+^ were transfected into Huh7 cells. HBV replication intermediates and mRNAs were further examined by Southern and northern blotting, respectively. Results showed that reinstated preS2 start codon did not change the replication and 3.5 kb pgRNA transcriptional levels of B56^M+^ in Huh7 cells (Figure 5c). Additionally, B56^M+^ was transfected into Huh-7 cells, resulting in significant expression level of HBsAg but not HBeAg compared to that with B56 (Figure 5d). The restoration of MHBs expression improving HBsAg secretion level was also observed in HepG2-NTCP cells which were inoculated with B56^M+^ virions (Figure S1).

To further test the effect of HBsAg expression on HBV persistence in mouse model, B56 was further modified to only lack LHBs (B56^M+/L-^), SHBs (B56^M+/S-^) and total HBsAg (B56^LMS-^). As shown in Figure 5e, the loss of LHBs or SHBs, or even the total disappearance of HBsAg in serum, also did not impair the persistence of B56 in HDI mice. Finally, B56, B56^M+^, B56^M+/L-^, B56^M+/S-^, and B5^LMS-^ were transfected into Huh7 cells. Results showed that B56^M+^ exhibited high production of HBsAg, and B56^M+/S-^ and B5^LMS-^ indeed lacked the presence of HBsAg in cell culture (Figure 5f). These results revealed that reduction or even deletion of HBsAg, to some extent, contributed to the persistence of HBV gene expression at an earlier stage in B56 HDI-mice.

### MHBs loss has no direct impact on the persistence of new HBV1.3 clone B6-PLUS

**Figure 6.**
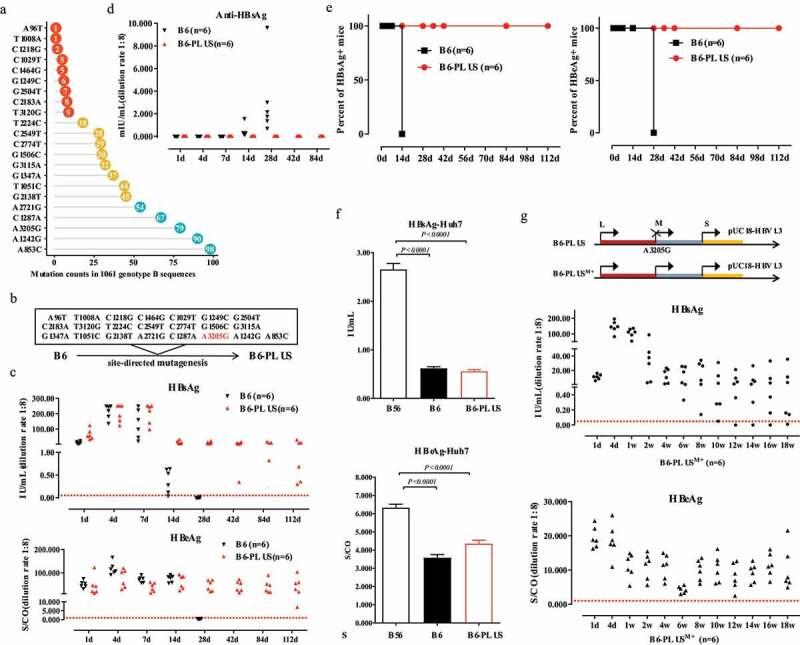
**Specific Mutations in B56 Contribute to the HBV Persistence in Mice**. (a) Twenty-two specific mutations in B56 with lower frequency were identified by the alignment of B56 with B6 and HBV genotype B isolates from NCBI GenBank. The numbers within the round circle show the frequency of that mutation. (b) The new clone B6-PLUS was generated based on B6 as backbone harboring these 22 specific mutations by site-directed mutagenesis as shown in the schematic illustration. The A3205G mutation led to start codon change (ATG to GTG) resulting in MHBs loss. Sera from B6 and B6-PLUS HDI mice were analyzed for HBsAg, HBeAg (c) and anti-HBsAg titers (d). (e) Positive rates of serum HBsAg and HBeAg in mice were analyzed. (f) Huh7 cells cultured in 24-well plates were transfected with B56, B6, and B6-PLUS. After 48 h of transfection, the culture supernatants were collected to detect HBsAg and HBeAg levels. (g) Schematic illustrations of the genomic (B6-PLUS, B6-PLUS^M+^). The HBsAg and HBeAg levels in mice induced by the hydrodynamic injection of B6-PLUS^M+^ with the re-instated preS2 start codon

Although B56 and B6 both belong to genotype B, only B56 persists long-term in BALB/c. Thus, it was speculated that some specific differences in the sequence determine B56 persistence. To confirm this, we compared B56 with B6 and other genotype B complete sequences from NCBI GenBank-registered sources through alignment of 1061 sequences (Figure 6a, details shown in Table S2). There were nucleotide differences between B56 and B6 (details shown in Table S3), among which, 22 mutations with a lower frequency in B56 were identified (Figure 6b).

It is difficult to study a single mutation because of the highly compact nature of the HBV genome, in which one nucleotide change also affects other related proteins. For this reason, a new pUC18-HBV1.3 plasmid (B6-PLUS) harboring all these 22 mutations was created based on B6 as a template. B6-PLUS also lacked the preS2 start codon because of the A3205G mutation (Figure 6b). As expected, the HDI of B6-PLUS resulted in sustained HBsAg and HBeAg expression in mice for over 112 days (Figure 6c). Serum negative for anti-HBsAg in B6-PLUS HDI-mice was in total contrast to the quick clearance of B6, which showed undetectable serum HBsAg with seroconversion to anti-HBsAg (Figure 6d). In addition, B6-PLUS persisted for 112 days in 100% of mice (n = 6; Figure 6e). Moreover, B6-PLUS displayed significant lower levels of HBsAg and HBeAg compared to B56 in transfected Huh7 cells (Figure 6f). As shown in Figure 6g, MHBs expression was restored in B6-PLUS^M+^-injected mice, which still led to the sustained expression of antigens for over 18 weeks. These results suggested that lower expression levels of HBsAg or the retention of MHBs does not affect B6-PLUS persistence in the mouse model, compared to that with B56.

### Cytokines CXCL1, CXCL2, IL-6 and IL-33 are elevated in B56^M+^ HDI-mice

**Figure 7.**
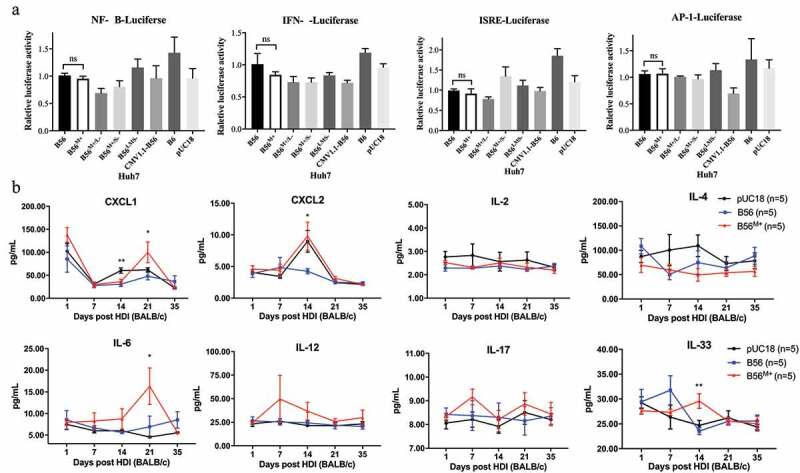
**CXCL1/CXCL2, IL-6 and IL-33 Are Associated with B56 ^M+^ Clearance in Mice**. (a) Huh7 cells in 24-well were transfected with 0.3 μg of HBV expressing plasmids or pUC18 vector, together with 0.15 μg of luciferase reporter plasmids (pNF-κB-Luc, pIFN-β-Luc, pISRE-Luc and pAP-1-Luc) and 0.1 μg of pPRL-TK for 48 h, respectively. The cell lysates were harvested and luciferase reporter activity was determined by dual luciferase reporter assays. (b) Serum levels of cytokines were quantitated using multiplex-based assay. Dynamic changes of CXCL1, CXCL2, IL-2, IL-4, IL-6, IL-12, IL-17 and IL-33 were profiled in pUC18-injected mice, B56-injected mice and B56^M+^-injected mice at 1, 7, 14, 21, 35 days. ns, there was no significant difference; *P < 0.05; **P < 0.01

Given that host defensing against viruses depended on the activity of cellular innate immune system, we firstly examined the promoter activations of NF-κB, AP-1, INF-β and interferon stimulation response element (ISRE) in transiently HBV-transfected Huh7 cells. The results of dual-luciferase reporter assay showed that only loss of B56 MHBs did not affect the activity of cellular innate immune response through NF-κB, INF-β, ISRE or AP-1 mediated signaling pathways (Figure 7a). To further gain insight into the immuno-reactivity to B56 persistence with or without MHBs loss, 11 cytokines (CXCL1, CXCL2, IL-2, IL-4, IL-6, IL-10, IL-12, IL-17, IL-21, IL-33 and TNF-α) serum were measured in mice injected with pUC18 or B56 or B56^M+^ at different time points (1, 7, 14, 21, 35 days). IL-10, IL-21 and TNF-α below the lower limits of detection were excluded from further analysis. As shown in Figure 7b, the levels of IL-2, IL-4, IL-12 and IL-17 were all not found to be significant difference in mice injected with pUC18, B56 and B56^M+^ at each time point. CXCL1 in B56^M+^-injected mice manifested an elevation (99.83 pg/mL± 44.98 pg/mL), which was higher than that in B56-injected mice (48.04 ± 17.94 pg/mL, *P* = 0.035) at day 21. Additionally, at day 14, CXCL2 in B56-injected mice (4.25 ± 0.91 pg/mL) was apparently lower than that in pUC18-injected mice (8.98 ± 3.79 pg/mL, *P* = 0.027) and B56^M+^-injected mice (9.81 ± 4.92 pg/mL, *P* = 0.038). These data suggested that restoration of MHBs (B56^M+^) induced CXCL1/CXCL2 level elevating. Interestingly, high peak serum IL-6 level (16.31 ± 9.46 pg/mL) was only observed in day 21 after injection of B56^M+^. In case of IL-33, it was obviously elevated at 14 days in B56^M+^-injected mice displaying rapid clearance, comparing with the persistence of B56-injected mice (*P* = 0.005) and pUC18-injected mice (*P* = 0.021).

## Discussion

To our knowledge, all mammalian hepadnaviruses contain MHBs, which is highly conserved within the specific N glycosylation motif [6–9]. However, the impact of MHBs on chronic HBV infection remains unclear. Here, we aimed to establish the role of MHBs in HBV persistence in HDI-mouse models. B56, a MHBs-deficient variant of HBV genotype B, was isolated from a CHB patient and recreated as a plasmid by cloning an HBV genome with a ~1.3-unit length into the pUC18 vector [20]. This clone displayed viral persistence in BALB/c mice for more than 70 weeks in our study, by contrast to mice injected with B6 as a control which showed a rapid HBsAg clearance and HBsAb seroconversion similarly to what is observed in acute self-resolving infections in humans. Moreover, reinstating the preS2 start codon in B56 resulted in the rapid clearance of B56^M+^ from mice within 28 days. Conversely, the loss of MHBs expression (B6^M-^) did not result in viral persistence in mice, indicating that MHBs loss is only one of the factors determining the persistence of HBV viral genome in this model.

The final outcome of HBV infection is dynamic interplay between host immune responses and viral factors such as viral genetic variation and genotypes [24]. The promoters and enhancers of B56 could be employed to create new genotype (A, B, C, and D) clones and promote their existence in the mouse model. These results demonstrated that promoters and enhancers of B56 guarantee the unique biological function and chronicity of B56. Of importance, the injection of pCMV-HBV1.1-B56 resulted in rapid and complete clearance in mice, further suggesting that the unique sequence of B56 with a 1.3-fold increase in genome length is the prerequisite for its chronicity.

Deletion of HBsAg is rare in the clinic [25]; nevertheless, HBV mutants carrying deletions in preS1/S2 were observed more frequently in patients with CHB or acute fulminant hepatitis [10,26], which indicates that these variants played a pathogenic role in the progression of HBV infection. This study only focused on the start codon mutation leading to MHBs loss rather than more combine mutations or deletions in preS2 which may produce truncated antigen proteins. LHB loss, SHBs loss, or even an entire HBsAg deletion did not impair B56 persistence, suggesting that the formation of HBV virions or SVPs was not the determining factor for B56 persistence in this mouse model. Even though the expression of MHBs is not essential for HBV replication, virion morphogenesis and secretion, this does not preclude the importance of MHBs retention early in HBV infection. Our observations revealed that, by contrast with LHBs and SHBs, MHBs-deficiency variants frequently presented in HBV chronic infection and the loss of MHBs due to start codon mutation was more closely associated with CHB patients than AHB patients. This was consistent with a study found that preS2-defective HBV variants were associated with active viral infection [27], suggesting that MHBs-deficient variants might survive as dominant strains which maintained lower HBsAg expression level, the result of host immune selection in HBV persistence infection.

Moreover, recent reports and our current study showed that abolishing MHBs expression was able to reduce the secretion level of total HBsAg [28,29]. A unique feature of MHBs is the N-linkage glycosylation site at the fourth position of the pre-S2 domain, which is not present in the S domain and is not used in the L protein due to cytoplasmic distribution. It was proposed that MHBs can improve the efficiency of HBV virions formation and secretion by contacting with L and S proteins at M-specific glycosylation site [30,31]. Conversely, non-glycosylated aberrant M protein acts in a dominant negative manner, disrupting the viral envelope and impeding HBV secretion [32]. 56B1.3 ^M-^ which was based on B6 as backbone and replaced by the specific promoters and enhancers from B56 had relatively lower levels of HBsAg and exhibited delayed clearance in mice. These results demonstrated that MHBs deletion might specifically contribute to HBV persistence in B genotype clones. MHBs loss might be a special strategy to reduce total HBsAg levels for HBV.

This speculating did not negate the conclusion that baseline higher HBsAg levels were associated with viral persistence in CHB patients who had a large HBV quasispecies pool. However, our investigation and results were only based on a special MHBs deficiency isolate that was caused by start codon mutation and an even more rare presentation in CHB patients as the dominant strain. Under the long-term response to host immune pressure, some surviving HBV strains are likely to express lower HBsAg levels, accompanied by MHBs deletion and adaptive mutations. If the MHBs expression is restored, it is possible to destroy the advantage of evolutionary escape and makes it easier to be eliminated, which is need to be further investigated by the detailed analysis with larger sample size to characterize the HBsAg levels in CHB patients carrying MHBs deficiency variants.

The new HBV1.3 clone termed B6-PLUS harboring these 22 specific mutations was reconstructed based on a B6 backbone and manifested as a chronic viral clone in the mouse model. At the functional level, B6-PLUS and B6 exhibited similarly lower HBsAg expression levels in Huh7 cells compared to those with B56. Of note, B6-PLUS with restored MHBs expression (B6-PLUS^M+^) and lower HBsAg expression levels also persistently existed in BALB/c mice for more than 18 weeks, inconsistent with the recovery of MHBs expression, which hampered B56 persistence. Obviously, it is accepted that a lack of MHBs alone cannot fully establish a chronic HBV-mice model, because B56 persistence in BALB/c was the result from multiple factors including specific mutations. For persistent expression in mice such as with B56, we hypothesized that a higher HBsAg expression level might be detrimental to the persistence of HBV gene expression, as reducing or even complete ablating HBsAg did not prevent B56 persistence. B56^M+^ was transfected into Huh7 cells and showed a much higher HBsAg expression level than B56. MHBs loss in B56 reducing the total HBsAg levels promoted its persistence in a mouse model. This effect was probably very small in B6-PLUS and B6-PLUS^M+^ because of the lower expression levels of HBsAg, compared with the high HBsAg expression level in B56.

After viral infection, innate immune receptors are able to detect the invading virus and subsequently initiate the synthesis of IFN and protective cellular genes via NF-κB signaling pathway to directly limit viral replication [33,34]. And the truncated MHBs protein was proved to be able to trigger the activation of AP-1 and NF-κB in transgenic mice [35]. However, we found that the presence of MHBs protein had no effect on the activation of ISRE, IFN-β, NF-κB and AP-1 mediated signaling pathway in transiently HBV-transfected Huh7 cells, suggesting the possibility of MHBs-mediated immune modulation through other signaling pathways during B56^M+^ clearance to be explored in further work. In addition, cytokine profile analysis revealed that CXCL1, CXCL2, IL-6 and IL-33 transient elevations were associated with restoration of MHBs expression in BALB/c mouse model, leading to HBV variants clearance. These results were in agreement with the report that HBV could evade innate immunity of hepatocytes but induce cytokine productions [36]. For viral infection, IL-6 signal is very important to control virus expansion by stimulating CD4+ T cells, leading to germinal center activation and improving antibody response in mice [37]. During HBV infection, IL-6 is secreted by the pro-inflammatory macrophage, which strongly inhibits the establishment of HBV infection in hepatocytes [38]. IL-8 has well-defined immunoregulatory effects on T cell function and inflammation and is an important mediator of innate immunity. Mouse chemokine CXCL1/cytokine inducing neutrophil attracting factor (KC), CXCL2/macrophage inflammatory protein 2 (MIP-2) are considered to be surrogates of human IL-8 which also play a key role in the immune pathogenesis of HBV infection [39,40]. IL-33 may activate follicular helper T cells, leading to promote humoral responses to HBV during the pathogenesis and induce HBV clearance in the mouse [20,41]. Thus, these cytokines may have protective roles in HBV infections and can be taken into account when clinic therapeutic strategy is considered.

Another potential limitation of this research is that HBV clones containing a 1.3 unit-length HBV genome were artificially generated and delivered into hepatocytes. This is quite different from real HBV infecting cells and may lead to the limited results. However, to date, neither natural nor genetically modified mice models can be naturally infected with HBV and used for in vivo study.

Taken together, the importance of MHBs for hepatitis B vaccine development has been emphasized in clinical trial [42]. Although disease activity and monitoring of treatment response in CHB patients can be predicted through the quantification of HBsAg [25,43,44], the biological function of MHBs remains largely unknown. Our study investigated the potential role of MHBs in HBV persistence and demonstrated that MHBs loss resulting from start codon mutation promoted HBV persistence in a mouse model and is frequently associated with CHB in patients. A better understanding of MHBs contribution to HBV persistence will provide new insight in the pathogenesis of CHB and for future therapeutic purposes.

## Supplementary Material

Supplemental Material

## Data Availability

The authors declare that all data supporting the findings of this study are available within the paper and its supplementary materials.
