# Supplementary material for: Hepatitis B virus middle surface antigen loss promotes clinical variant persistence in mouse models
Source: Virulence. 2021 Nov 21;12(1):2868–82. doi: 10.1080/21505594.2021.1999130 (PMC8632123; doi:10.1080/21505594.2021.1999130)
Supplement: Supplemental Material [file KVIR_A_1999130_SM4769.docx]

**Supplementary Table 1. Sequences with MHBs loss due to start codon mutations from three CHB patients**

| **Rate of loss** | **Mutations in start condon** | |
| --- | --- | --- |
|  | **ATA** | **GTG** |
| MHBs loss (11/150, 7.33%) | KU964218 (Patient: 2403; Genotype: C) | KU964166 (Patient: 2529; Genotype: B) |
|  | KU964222 (Patient: 2403; Genotype: C) |  |
|  | KU964176 (Patient: 2441; Genotype: C) |  |
|  | KU964174 (Patient: 2441; Genotype: C) |  |
|  | KU964184 (Patient: 2441; Genotype: C) |  |
|  | KU964183 (Patient: 2441; Genotype: C) |  |
|  | KU964181 (Patient: 2441; Genotype: C) |  |
|  | KU964178 (Patient: 2441; Genotype: C) |  |
|  | KU964182 (Patient: 2441; Genotype: C) |  |
|  | KU964179 (Patient: 2441; Genotype: C) |  |
| LHBs loss （0/150, 0.00%） | / | / |
| SHBs loss （0/150, 0.00%） | / | / |

ALT: Alanine aminotransferase; LHBs: Large hepatitis B surface proteins; MHBs: Middle hepatitis B surface proteins; SHBs: Small hepatitis B surface proteins

**Supplementary Table 2. Full-length sequences with MHBs loss, LHBs loss or SHBs loss registered in NCBI GenBank.**

|  | **MHBs loss (126/1061, 11.88%)** | | | | | | | | | **LHBs loss （1/1061, 0.09%）** | **SHBs loss （0/1061, 0.00%）** |
| --- | --- | --- | --- | --- | --- | --- | --- | --- | --- | --- | --- |
| **Mutations in start condon** | **ACA** | **GAG** | **CTG** | **GTC** | **ACG** | **ATT** | **GTA** | **ATA** | **GTG** | **ACG** | **/** |
| **GenBank ID** | AY217375.1 | KJ803775.1 | AB819613.1 | JQ429079.1 | AB241117.1 | EU570075.1 | JN792901.1 | AB073837.1 | AB205122.1 | AB368295.1 | / |
|  |  |  | KP406311.1 | JQ429080.1 | FJ032342.1 | EU579441.1 | JN792902.1 | AB073842.1 | AY206373.1 |  |  |
|  |  |  |  |  | JQ027315.1 | EU589335.1 | KP406180.1 | AB287318.1 | DQ463793.1 |  |  |
|  |  |  |  |  | KT991433.1 | EU939660.1 | KP406186.1 | AY217361.1 | DQ463801.1 |  |  |
|  |  |  |  |  |  | FJ032344.1 | KP406187.1 | AY217376.1 | DQ993682.1 |  |  |
|  |  |  |  |  |  | FJ386608.1 | KP406188.1 | AY220697.1 | EU939633.1 |  |  |
|  |  |  |  |  |  | FJ386656.1 | KP406189.1 | EF494381.1 | EU939634.1 |  |  |
|  |  |  |  |  |  | LC064372.1 | KP406191.1 | EU939629.1 | EU939670.1 |  |  |
|  |  |  |  |  |  |  | KP406192.1 | EU939631.1 | EU939671.1 |  |  |
|  |  |  |  |  |  |  | KP406193.1 | EU939639.1 | JN792893.1 |  |  |
|  |  |  |  |  |  |  | KP406194.1 | GQ924625.1 | JN792894.1 |  |  |
|  |  |  |  |  |  |  | KP406195.1 | GQ924646.1 | JQ027334.1 |  |  |
|  |  |  |  |  |  |  | KP406196.1 | HM011474.1 | JQ429081.1 |  |  |
|  |  |  |  |  |  |  | KP406197.1 | JF436921.1 | KJ803817.1 |  |  |
|  |  |  |  |  |  |  | KP406198.1 | JQ027325.1 | KJ843165.1 |  |  |
|  |  |  |  |  |  |  | KP406199.1 | KJ803755.1 | KP406176.1 |  |  |
|  |  |  |  |  |  |  | KP406209.1 | KJ803758.1 | KP406177.1 |  |  |
|  |  |  |  |  |  |  | KP406210.1 | KM213032.1 | KP406178.1 |  |  |
|  |  |  |  |  |  |  | KP406211.1 | KP406281.1 | KP406180.1 |  |  |
|  |  |  |  |  |  |  | KP406213.1 | KP406283.1 | KP406181.1 |  |  |
|  |  |  |  |  |  |  | KP406214.1 | KP406284.1 | KP406183.1 |  |  |
|  |  |  |  |  |  |  | KP406215.1 | KP406285.1 | KP406184.1 |  |  |
|  |  |  |  |  |  |  | KP406216.1 | KP406286.1 | KP406185.1 |  |  |
|  |  |  |  |  |  |  | KP406217.1 | KP406287.1 | KP406255.1 |  |  |
|  |  |  |  |  |  |  | KP406218.1 | KP406289.1 | KP406257.1 |  |  |
|  |  |  |  |  |  |  | KP406219.1 | KP406294.1 | KP406262.1 |  |  |
|  |  |  |  |  |  |  |  | LC064366.1 | KP406263.1 |  |  |
|  |  |  |  |  |  |  |  | LC064367.1 | KP406265.1 |  |  |
|  |  |  |  |  |  |  |  | MF674441.1 | KP406268.1 |  |  |
|  |  |  |  |  |  |  |  | MF674443.1 | KP406270.1 |  |  |
|  |  |  |  |  |  |  |  | MF674461.1 | KP406272.1 |  |  |
|  |  |  |  |  |  |  |  | MF674465.1 | KP406273.1 |  |  |
|  |  |  |  |  |  |  |  |  | KP406274.1 |  |  |
|  |  |  |  |  |  |  |  |  | KP406275.1 |  |  |
|  |  |  |  |  |  |  |  |  | KP406276.1 |  |  |
|  |  |  |  |  |  |  |  |  | KP406278.1 |  |  |
|  |  |  |  |  |  |  |  |  | KP406280.1 |  |  |
|  |  |  |  |  |  |  |  |  | KT749820.1 |  |  |
|  |  |  |  |  |  |  |  |  | KT991430.1 |  |  |
|  |  |  |  |  |  |  |  |  | KX276775.1 |  |  |
|  |  |  |  |  |  |  |  |  | KX276817.1 |  |  |
|  |  |  |  |  |  |  |  |  | KX276818.1 |  |  |
|  |  |  |  |  |  |  |  |  | KY470834.1 |  |  |
|  |  |  |  |  |  |  |  |  | KY470837.1 |  |  |
|  |  |  |  |  |  |  |  |  | KY470841.1 |  |  |
|  |  |  |  |  |  |  |  |  | KY470842.1 |  |  |
|  |  |  |  |  |  |  |  |  | MF674419.1 |  |  |
|  |  |  |  |  |  |  |  |  | MF674436.1 |  |  |
|  |  |  |  |  |  |  |  |  | MF674459.1 |  |  |
|  |  |  |  |  |  |  |  |  | MF674499.1 |  |  |

**Supplementary Table 3. The nucleotide sequence differences between B56, B6 and B6-PLUS**

|  |  | **Mutations** | **Located Regions** |
| --- | --- | --- | --- |
| **B6 vs B56** | **B6 vs B6-PLUS** | **A96T** | preS2 / Polymerase |
|  |  | **A853C** | Polymerase |
|  |  | **T1008A** | Enhancer I / Polymerase |
|  |  | **C1029T** | Enhancer I / Polymerase |
|  |  | **T1051C** | Enhancer I / Polymerase |
|  |  | **C1218G** | Enhancer I / X promoter / Polymerase |
|  |  | **A1242G** | Enhancer I / X promoter / Polymerase |
|  |  | **G1249C** | Enhancer I / X promoter / Polymerase |
|  |  | **C1287A** | Enhancer I / X promoter / Polymerase |
|  |  | **G1347A** | Enhancer I / X promoter / Polymerase |
|  |  | **C1464G** | X / Polymerase |
|  |  | **G1506C** | X / Polymerase |
|  |  | **G2138T** | preC / core |
|  |  | **C2183A** | preC / core |
|  |  | **T2224C** | preC / core |
|  |  | **G2504T** | Polymerase |
|  |  | **C2549T** | Polymerase |
|  |  | **A2721G** | S promoter 1 / Polymerase |
|  |  | **C2774T** | S promoter 1 / Polymerase |
|  |  | **G3115A** | S promoter 2 / preS1 / Polymerase |
|  |  | **T3120G** | S promoter 2 / preS1 Polymerase |
|  |  | **A3205G** | preS2 / Polymerase |
|  | **B6-PLUS vs B56** | G25T | preS2 / Polymerase |
|  |  | T52C | preS2 / Polymerase |
|  |  | A285G | S / Polymerase |
|  |  | T499A | S / Polymerase |
|  |  | C574A | S / Polymerase |
|  |  | A753T | S / Polymerase |
|  |  | A853C | Polymerase |
|  |  | G870T | Polymerase |
|  |  | A940C | Polymerase |
|  |  | G1005T | Enhancer I / Polymerase |
|  |  | G1032A | Enhancer I / Polymerase |
|  |  | A1149C | Enhancer I / Polymerase |
|  |  | C1221A | Enhancer I / X promoter / Polymerase |
|  |  | T1350C | Enhancer I / X promoter / Polymerase |
|  |  | G1386A | X / Polymerase |
|  |  | T1470C | X / Polymerase |
|  |  | C1632A | X / Enhancer II / Core promoter |
|  |  | T1638C | X / Enhancer II / Core promoter |
|  |  | A1752G | X / Enhancer II / Core promoter |
|  |  | C2101T | preC / Core |
|  |  | G2293A | preC / Core |
|  |  | T2526G | Polymerase |
|  |  | A2561G | Polymerase |
|  |  | C2627A | Polymerase |
|  |  | G2648A | Polymerase |
|  |  | C2712T | S promoter 1 / Polymerase |
|  |  | A2738G | S promoter 1 / Polymerase |
|  |  | A2739C | S promoter 1 / Polymerase |
|  |  | T2771G | S promoter 1 / Polymerase |
|  |  | A3042G | S promoter 2 / preS1 / Polymerase |
|  |  | A3097C | S promoter 2 / preS1 / Polymerase |
